# Intranasal methylene blue administration confers neuroprotection in rats subjected to exhaustive exercise training

**DOI:** 10.3389/fnbeh.2025.1648837

**Published:** 2025-09-23

**Authors:** Guangcong Peng, Wenxin Pan, Ziheng Cai, Long Lin, Xu Ma

**Affiliations:** ^1^Key Laboratory of Brain, Cognition and Education Sciences, Ministry of Education, Institute for Brain Research and Rehabilitation, and Guangdong Key Laboratory of Mental Health and Cognitive Science, South China Normal University, Guangzhou, China; ^2^Nanfang College Guangzhou, Guangzhou, China

**Keywords:** exhaustive exercise, methylene blue, intranasal administration, mitochondria, neuroprotection

## Abstract

**Introduction:**

Emerging evidence suggests that exercise-induced fatigue negatively affects nervous system function, yet effective mitigation strategies are limited. This study aimed to determine whether intranasal methylene blue (MB) could prevent neurological deficits induced by exhaustive exercise in a rat model.

**Methods:**

We utilized a rat exhaustive exercise training paradigm. Animal body weight was monitored, and a battery of behavioral tests was conducted to evaluate locomotor activity, anxiety-like behaviors, and spatial learning and memory. At the cellular level, we assessed neuron loss, apoptosis, synaptic proteins, myelin sheath, gliosis, and mitochondrial morphology in the hippocampal CA1 region and the striatum.

**Results:**

Rats subjected to exhaustive exercise exhibited reduced locomotor activity, increased anxiety-like behaviors, and impaired spatial memory. This was associated with significant neuron loss, activation of apoptotic pathways, loss of synaptic proteins and myelin sheath, gliosis, and compromised mitochondrial morphology in the hippocampus and striatum. Notably, intranasal MB treatment significantly rescued these neuronal damages and improved performance in behavioral tests.

**Discussion:**

Our findings demonstrate the neuroprotective effects of intranasal MB against exhaustive exercise-induced neurological deficits. This suggests that MB is a promising therapeutic agent for preventing the adverse neurological consequences of extreme physical exertion.

## Graphical Abstract

**Figure:**
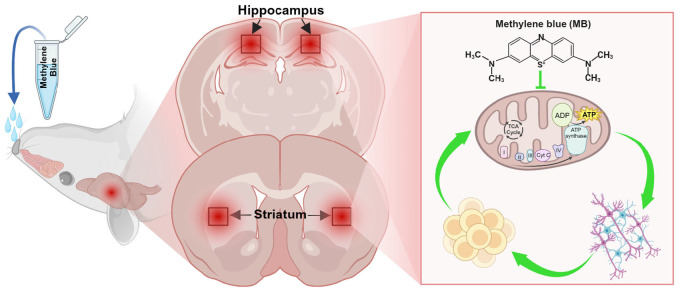
Intranasal methylene blue (MB) provides neuroprotection against exhaustive exercise-induced deficits by enhancing mitochondrial function and rescuing behavioral performance.

## 1 Introduction

Exercise-induced fatigue is typically caused by prolonged duration or excessive intensity of physical activity. Based on the affected area, it can be categorized into peripheral fatigue and central fatigue (Carroll et al., 2017). Peripheral fatigue not only leads to muscle damage and increased cardiopulmonary stress but also results in bone and joint injuries (Shaktivesh et al., 2020). Additionally, exercise-induced fatigue involves central fatigue, which encompasses various mechanisms, including mitochondrial dynamics imbalance, neuronal apoptosis in certain brain regions, synaptic plasticity impairment, and inflammatory responses. Besides its adverse effects on athletic performance, prolonged exhaustive exercise has been shown to induce neurological deficits, including proprioceptive imbalances, temporary behavioral abnormalities, and changes in cognitive function (Gandevia, 2001; Lou et al., 2023). This collection of symptoms, usually associated with overtraining syndrome, not only impairs athletes’ competitive performance but also affects their quality of life. Developing effective therapeutic strategies to mitigate the negative effects of prolonged exhaustive exercise, therefore, has become a key focus in the fields of sports medicine and neuroscience (Meeusen et al., 2013). Therefore, developing therapeutic strategies to mitigate the negative effects of prolonged exhaustive exercise on the central nervous system (CNS) is imperative (Jones et al., 2011).

After intense physical activity, blood circulation is redistributed, with skeletal muscles requiring more blood while the brain receives less, inevitably leading to cerebral F. This state following intense physical activity can result in damage to the CNS, including neuronal apoptosis and synaptic damage. Studies have revealed that after prolonged exhaustive exercise, certain brain regions in the CNS show increased expression of pro-apoptotic proteins like caspase-1, while expression of anti-apoptotic proteins is decreased, this triggers a cascade of responses in the caspase family of proteases, ultimately leading to apoptosis (Zhang et al., 2016; Han et al., 2018). Mitochondria play a crucial role in cellular energy supply and oxidative stress regulation. Maintaining mitochondrial dynamic balance involves processes such as mitochondrial fission, fusion, and autophagy (Zou et al., 2022; Singh and Singh, 2025). Highly dynamic mitochondria constantly oscillate between fission and fusion states, with the equilibrium achieved through these processes determining their morphology. Prolonged exhaustive exercise damages mitochondrial function, resulting in abnormal synaptic plasticity and neurological deficits (Davis et al., 2009; Liu et al., 2020). Consequently, mitochondrial dysfunction is a key indicator of unfavorable outcomes following exercise-induced fatigue. Prolonged intense exercise can activate microglia and astrocytes, prompting the secretion of large quantities of inflammatory factors, further exacerbating neuroinflammation. Iba-1 is specifically expressed in activated microglia, playing a role in immune response and regulation of neuroinflammation. Iba-1 also interacts with synapses to maintain the normal structure and function of the CNS (Kodali et al., 2021; Kann, 2024). GFAP is expressed in astrocytes, which tend to shift from a resting state to an enlarged, hypertrophic state when the CNS is diseased or under pathological conditions. GFAP can serve as a marker for astrocyte proliferation following exercise-induced fatigue.

The hippocampus and striatum play significant roles in the CNS. Through their respective functional specializations, they work in tandem to support memory formation and behavioral regulation, jointly sustaining the advanced functions of the CNS (Albouy et al., 2008). The hippocampus, as part of the brain’s limbic system, is critical for various cognitive functions, particularly learning, memory, and emotional regulation (Pronier et al., 2023). The striatum, as part of the basal ganglia, is located deep within the forebrain and is primarily associated with motor control, rewards, motivation, and habit learning. It contributes to the selection of complex behaviors and habits through the architecture of the basal ganglia (Kreitzer and Malenka, 2008; Fang and Creed, 2024). Given the critical functional significance of the hippocampus and striatum, alterations in physiological processes in these regions may underlie the adverse neurological consequences resulting from prolonged exhaustive exercise.

Originally introduced as a dye, methylene blue (MB) is now widely studied and applied for its diverse biological and medical functions (Schirmer et al., 2011). The drug initially played a significant role in malaria treatment, and it was later discovered to have antipsychotic effects when used in patients with psychiatric disorders. From the 1930s to the 1990s, the antifungal, antiparasitic, and antibiotic properties of MB and its derivatives, as well as their ability to reverse neurotoxicity in encephalopathy, were successively discovered (Ohlow and Moosmann, 2011). Recent studies have shown that MB exhibits significant neuroprotective effects in various neurological disorders, such as cerebral ischemia, Alzheimer’s disease, Parkinson’s disease, and traumatic brain injury (Fenn et al., 2015; Li et al., 2018; Gureev et al., 2022). In summary, MB has demonstrated benefits in enhancing mitochondrial function, alleviating oxidative stress and inflammation, and improving cognitive functions in the CNS, and it has been utilized in clinical treatments and drug development. Meanwhile, intranasal administration allows small molecule drugs to bypass the blood-brain barrier and directly reach the brain via pathways like the olfactory and trigeminal nerves, enabling targeted CNS therapy. Given that MB is both hydrophilic and lipophilic, it can penetrate biological membranes effectively. Therefore, MB is well-suited for intranasal administration as a delivery method (Xue et al., 2021).

This study aims to investigate the effects of intranasal administration of MB on neuronal loss, synaptic plasticity damage, and mitochondrial dynamics in a rat model of exhaustive swimming exercise. In summary, this study aims to explore the neuroprotective effects of MB, providing a convenient approach to mitigate the negative impacts of exercise-induced fatigue.

## 2 Materials and methods

### 2.1 Animals and grouping

The rats used in this study were purchased from the Guangzhou Medical Laboratory Animal Center. The animal housing conditions were maintained at a temperature of 22 °C ± 2 °C, a humidity level of 60% ± 5%, with a normal light-dark cycle and quiet environment. After the rats arrived, all animals were housed in the animal facility and underwent a 14-days acclimatization period before any experimental procedures began. This critical stage aims to reduce stress caused by transportation and allow the animals to adapt to the new housing environment (constant temperature and humidity, 12-h light-dark cycle). During this period, the rats have free access to standard laboratory feed and water. The main purpose of this acclimatization period is to ensure that all animals reach a stable physiological and behavioral phenotype, thereby minimizing confounding variables and ensuring the validity of subsequent experimental data. After a 14-days acclimatization period, healthy 2-months-old male Sprague-Dawley (SD) rats were randomly divided into three groups: (a) the naive control group, which remained in a water environment without swimming (control group, *n* = 6), (b) the exhaustive exercise training group (EE, *n* = 7), and (c) the group treated with intranasal MB following exhaustive exercise training (EE + MB, *n* = 7). All animals involved in this experiment received approval from the Institutional Animal Care and Use Committee of South China Normal University and complied with the established animal welfare guidelines.

### 2.2 Exhaustive swimming training

**FIGURE 1:**
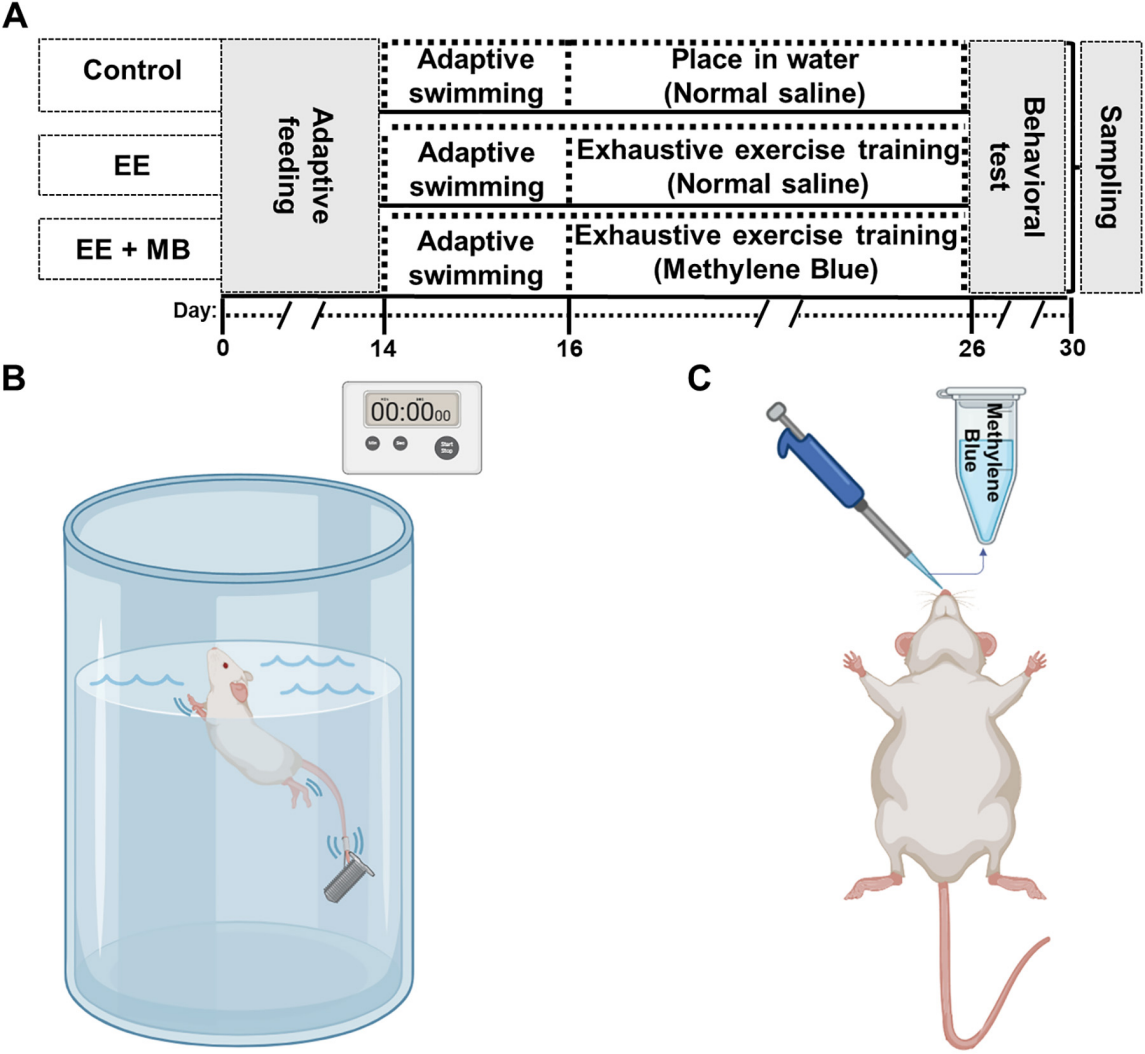
Experimental timeline diagram, establishment of exhaustive exercise model and methylene blue administration schematic. **(A)** Rats underwent exhaustive exercise training for ten consecutive days (from day 16 to day 26), followed by MB nasal administration in the MB group after each training session. Behavioral tests were conducted from day 26 to day 30, and immediately after the tests, the animals were sacrificed to extract the whole brain for further analysis. **(B)** A schematic diagram of exhaustive swimming training in rats, with a timer used to measure the time it takes for the animals to reach a state of exhaustion during swimming. **(C)** Schematic of MB administration via the nasal cavity in rats. EE, exhaustive exercise; MB, methylene blue.

The exhaustive swimming training was performed as described previously with slight modifications (Pierce et al., 1984; Peng et al., 2021). The experimental design is illustrated in Figure 1A. This study involved three experimental procedures for the animals: adaptive swimming phase, exhaustive swimming training, and behavioral testing. As shown in Figure 1B, the swimming training was conducted in a 55 cm-diameter bucket filled with water to a depth of 50 cm. The water temperature was maintained at 30 °C ± 2 °C. To minimize water-induced stress on the animals, the rats underwent a 3-days adaptive swimming training before the experiment, with swimming durations of 10 min, 15 min, and 20 min on the first, second, and third days, respectively. During adaptive swimming, we observe the animals’ condition after swimming, and proceed with swimming training if no obvious abnormalities are detected. Following the adaptive swimming training, the rats underwent exhaustive swimming training for 10 consecutive days. During this phase, weights equivalent to 3% of body weight were attached to the animals’ tails, and the training lasted at least for 2 h daily, from 5:00 PM to 11:00 PM. To prevent the rats from floating or becoming overly stressed during swimming, a stationary brush was used to stir the water gently and continuously. To objectively and accurately determine fatigue points, we adopted a multi-component “Fatigue State Score,” which was refined based on our previous work (Peng et al., 2021). An animal was judged to have reached exhaustion, and its trial was immediately terminated, upon accumulating a cumulative score of two or more based on the following criteria, with each point assigned upon its first definitive observation: (1) Critical Submersion, defined as the animal remaining fully submerged for a continuous 10 s; (2) Loss of Coordinated Motor Control, characterized as a composite of inefficient and abnormal swimming postures such as pronounced upper-body torsion, a significant backward tilt from the normal swimming angle, or a clear shift to frantic and ineffective forelimb paddling; and (3) Onset of Sustained Vocalization, defined as the animal beginning to emit continuous and clearly audible “squeaking” sounds. This objective scoring system ensures a reproducible and ethically-sound endpoint for the determination of fatigue. Meanwhile, the control group animals were placed in shallow water environment (5 cm deep) at the same temperature for the same duration.

### 2.3 Methylene blue nasal administration

The dose and route of administration of MB were based on a previous study (Zhang et al., 2020; Peng et al., 2021). In brief, a 1% MB solution (10 mg/ml) was prepared by dissolving and diluting MB in 0.9% saline. As shown in Figure 1C, 1 h after each exhaustive swimming training session, rats were anesthetized with vaporized isoflurane and positioned on their backs. The MB solution (10 mg/ml) was administered intranasally using a pipette in a dropwise fashion (2.5 μL/drop), alternating between the left and right nostrils every 2–3 min. Approximately 10 μL was delivered into each nostril, with a total administration volume of about 20 μL per session. Meanwhile, rats in both the Control (sedentary) and the EE groups were administered an identical volume of the vehicle (0.9% saline) using the same intranasal administration protocol.

### 2.4 Monitoring animal weight and behavioral testing

The body weight of rats was measured throughout the exhaustive exercise training procedure. All rats underwent behavioral testing in a quiet environment with controlled light intensity after the final session of exhaustive exercise and MB administration. The order of the tests was as follows: the open field test (day 26), the elevated plus maze test (day 27), and the Barnes maze test (day 27–30). Before testing, the rats were acclimated to the laboratory for at least 4 h.

### 2.5 Open field test

The open field test is commonly used to assess animals’ exploratory behavior, locomotor ability, and anxiety levels (Zhao et al., 2024). In this experiment, a wooden open field box with dimensions of 60 cm in length, 60 cm in width, and 45 cm in height was used. The black floor of the box was evenly divided into nine equal squares with white paint. In a quiet environment, rats were placed in the open field and allowed to move freely for 5 min, while their movement trajectories were recorded and analyzed using ANY-maze software. After the test, 75% ethanol was used to clean the open field to eliminate any odors.

### 2.6 Elevated plus maze (EPM)

The elevated plus maze is a classic method used to evaluate anxiety-like behavior in rodents, based on their conflicting tendencies to explore elevated open spaces and their fear of such environments (Wu et al., 2020). At the start of the experiment, rats are placed in the central area of the maze and allowed to move freely for 5 min. The number of entries into the open arms and the time spent in the open arms are negatively correlated with anxiety levels. After testing each animal, the maze platform is wiped thoroughly with 75% ethanol three times, and then allowed to dry for 5 min to remove any residual odors.

### 2.7 Barnes maze

The Barnes maze experiment is a method used to assess spatial learning and memory in animals, as previously described by our laboratory (Yang et al., 2022). This experiment utilizes a black circular maze platform with 18 holes, one of which (located at the 2:00 position) has a black escape box underneath (13 cm × 10 cm × 11 cm). Rats undergo 3 days of spatial learning and memory training followed by 1 day of spatial memory testing. Training trial (Days 27–29): before training begins, one of the holes on the maze is designated as the target hole, with a black target box placed underneath it. Bright lighting serves as a stimulus, and the rats are allowed to freely explore the maze within a set time frame to locate the target box. Spatial memory testing (Day 30): On the probe test day, the black escape box was removed, and a camera recorded the rats’ exploration paths on the platform for 90 s. The recorded data are analyzed using any maze video tracking software (Stoelting Co., Wood Dale, IL, USA). After each test, the platform is cleaned with 75% ethanol and air-dried. At the end of the testing, the time spent exploring the target zone is quantified to evaluate learning and memory performance.

### 2.8 Sample collection

Following the behavioral testing, the animals were anesthetized and sampled as quickly as possible. In brief, after anesthetizing the animals with isoflurane, cardiac perfusion was carried out using ice-cold physiological saline. The brain tissues were removed promptly and divided into two halves. From one hemisphere, the hippocampus and striatum were dissected and preserved at −80 °C for protein preparation. The other hemisphere was fixed overnight in 4% paraformaldehyde, then dehydrated in 30% sucrose solution until it sinks (Yang et al., 2019). Subsequently, the dehydrated brain tissues were embedded in OCT freezing compound and frozen at −80 °C overnight. Finally, 20-μm-thick coronal brain sections were prepared using a Leica cryostat (Leica Biosystems CM1850).

### 2.9 Cresyl violet staining

Cresyl violet staining was used to quantify the number of surviving neurons. Briefly, 3–5 brain slices were randomly selected from each animal, rinsed with PBS for 30 min, mounted on slides, and air-dried. The slices were stained using tar violet staining solution for 12 min and then heated in a 37 °C incubator. Gradual differentiation was performed as follows: decolorization in deionized water for 20 s, followed by sequential alcohol washes (70%, 80%, 90%, and 100%) for 3–4 s each. The slices were fixed with xylene for 5 min before coverslipping. A microscope was used to observe neuron density, capture images, and analyze the number of surviving neurons in the hippocampal CA1 pyramidal layer. Surviving neurons were identified by their rounded shape and distinctly stained nuclei, while abnormal or dead neurons appeared abnormally condensed and aggregated.

### 2.10 Immunofluorescence staining

Immunofluorescence staining was performed as in our previous study (Wu et al., 2018). Briefly, floating brain sections (20 μm) were incubated with 10% normal donkey serum at room temperature for 1 h, followed by overnight incubation with the appropriate primary antibodies. The primary antibodies used in this study were as follows: anti-NeuN (1:100, Abcam), anti-synaptophysin and spinophilin (1:100, Cell Signaling), anti-Iba1 (1:200, Abcam), anti-GFAP (1:200, Cell Signaling), anti-MBP (1:100, Abcam), anti-Annexin V (11060-1-AP, Proteintech), anti-cleaved Caspase-3 (1:300, CST) and anti-TOM20 (11802-1-AP, Proteintech). After 24 h of primary antibody incubation, brain sections were washed three times with 0.4% Triton for 40 min each time, followed by 2 h of incubation in the dark with matched Alexa Fluor donkey anti-mouse/rabbit secondary antibodies (555/488, Thermo Fisher). The sections were then stained for apoptosis using a TUNEL assay kit (Beyotime, C1088) and subsequently mounted and coverslipped using Vectashield mounting medium containing DAPI (Solarbio, China). More than one area per brain was quantified: 3 areas in the CA1 region/striatum were evenly selected on each slice, and 3 – 4 slices were chosen from each rat.

### 2.11 Statistical analysis

All statistical analyses were performed using SigmaStat software (Systat Software; San Jose, CA, USA). All datasets passed the normality test. For comparisons among three groups at a single time point, one-way analysis of variance (ANOVA) followed by Tukey’s *post-hoc* test was used. For data involving repeated measurements over time, such as daily body weight, a two- way ANOVA analysis followed by the Tukey’s multiple comparisons was performed to assess the effects of group and time, as well as their interaction. The Student’s *T*-test was used to analyze data between two groups. All data were presented as mean ± standard error (SE). *P*-values were calculated using GraphPad Prism 8 and SigmaStat 3.5 software. *P*-values ≤ 0.05 are considered statistically significant.

## 3 Results

### 3.1 MB improved time to exhaustion and physical condition of rats exposed to exhaustive exercise training

**FIGURE 2:**
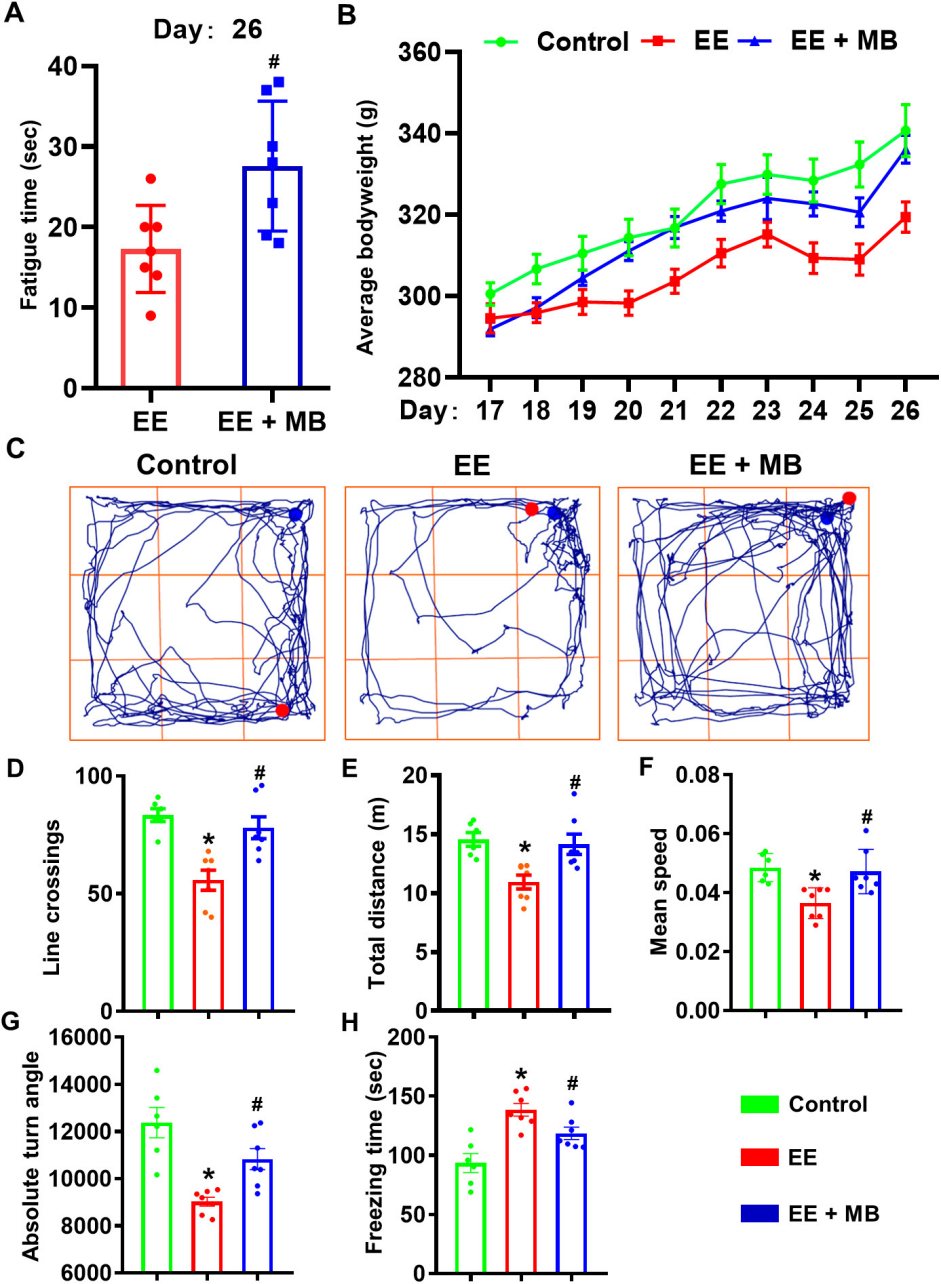
Methylene blue (MB) improved time to exhaustion and physical condition of rats exposed to exhaustive exercise training. **(A)** On day 26, the time until exhaustion for rats in the EE group and MB group during swimming was recorded. **(B)** The weight gain changes in the three groups of rats from day 17 to day 26. **(C)** The schematic of the open field test (day 26). The movement trajectories of each group. **(D)** The number of line crossing of the rats in the open field test. **(E)** The total distance traveled of the rats during the open field test. **(F)** The average speed of the rats in the open field test. **(G)** The absolute turning angle of the rats in the open field test. **(H)** The freeing time of the rats in the open field test. Data represent mean ± SE (*n* = 6–7). *P < 0.05 versus control group, #*P* < 0.05 versus EE group. Statistical significance was determined using two-tailed *t*-tests, One-way ANOVA analysis followed by the Tukey’s multiple comparisons or Two-way ANOVA analysis followed by the Tukey’s multiple comparisons.

Time of exhaustion can comprehensively reflect the degree of exercise-induced fatigue in rats (Chen et al., 2022). To investigate the effect of intranasal MB administration on exercise-induced fatigue in rats, we recorded the time it took for rats in the EE group and MB group to swim to exhaustion under a weighted load on the 26th day. The state of swimming exhaustion is defined as the point when the rats repeatedly sank, showed a significant decline in motor coordination, and remained submerged at the bottom of the water for over 10 s without returning to the surface. As shown in Figure 2A, compared to the EE group, the MB group demonstrated a significant increase in swimming time to exhaustion (*P* < 0.05). This indicates that 10 days of intranasal MB administration can reduce the degree of exercise-induced fatigue in rats and promote recovery from fatigue.

Weight changes during periods of exhaustion can indirectly reflect the health status of an organism, when an organism experiences significant fatigue, noticeable fluctuations in body weight can occur (Jeukendrup, 2011). To investigate the effect of intranasal administration of MB on the body weight of rats, we recorded changes in their weight. As shown in Figure 2B, we observed the dynamic changes in body weight during the 10-days exhaustive swimming scheme. Visually, a trend emerged where the EE group exhibited lower average body weights compared to the control group, while the EE + MB group’s weight trended in between the two groups. A two-way repeated measures ANOVA was conducted to analyze these changes. The analysis revealed a significant main effect for time (F (1.186, 20.15) = 141.9, *P* < 0.0001), but the main effect for treatment group was not significant (F (2, 17) = 2.672, *P* = 0.0979). Crucially, there was a highly significant interaction effect between the treatment group and time (F (18, 153) = 4.044, *P* < 0.0001), indicating that the weight-change trajectories over the 10 days were significantly different among the three groups. However, subsequent *post-hoc* tests comparing the groups at each individual time point did not find statistically significant differences (all *P* > 0.05). This lack of significance at specific days, despite the overall differing trends, may be attributed to the notable individual variations within each group, as suggested by the error bars.

Therefore, while intranasal MB administration did not result in a statistically significant weight recovery on any single day within this experimental timeframe, the overall trend and the significant interaction effect suggest that MB may have a mitigating effect on weight loss induced by exhaustive exercise.

To investigate the effects of MB on the locomotor activity and exploratory behavior of rats exposed to exhaustive exercise training, the open field test was conducted 4 h after the last exhaustive swimming session. The representative movement trajectories of rats in each group are shown in Figure 2C. The results indicate that the activity level of rats in the EE group was reduced compared to the other two groups. Compared to the performance of the control group in the open field, the EE group exhibited a significant reduction in line crossings, movement distance, average movement speed, and absolute turning angles (*P* < 0.05, Figures 2D–G), along with a significant increase in freezing time (*P* < 0.05, Figure 2H). These findings suggest that after 10 days of exhaustive swimming, the overall locomotor and exploratory activity of the EE group rats were significantly lower than those of the control group, indicating a pronounced state of fatigue. In contrast, the MB group showed a remarkable improvement in activity levels compared to the EE group (*P* < 0.05, Figures 2D–G), with a significant reduction in freezing time (*P* < 0.01, Figure 2H). This indicates that intranasal administration of MB can promote recovery of exercise capacity in fatigued rats and enhance their activity levels.

### 3.2 MB improved anxiety-like behavior and spatial memory ability in rats exposed to exhaustive exercise training

**FIGURE 3:**
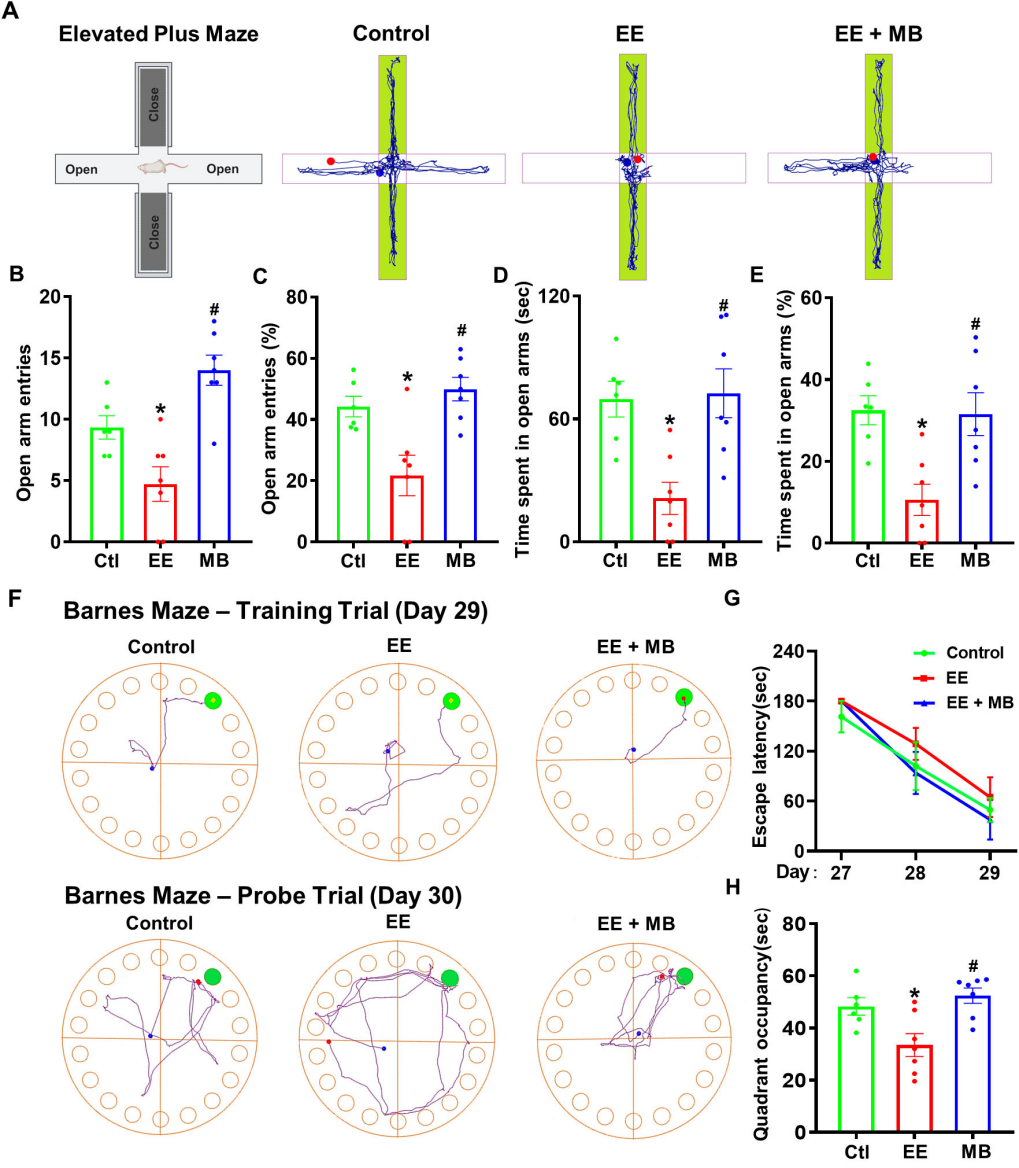
Methylene blue (MB) improved anxiety-like behavior and spatial memory ability in rats exposed to exhaustive exercise training. **(A)** Elevated plus maze (Day 27). Schematic of the elevated plus maze and representative activity diagrams of three groups of rats in the maze. **(B)** Number of times entered the open arm of the elevated cross maze. **(C)** The percentage (%) of rats entering the open arm of the elevated cross maze. **(D)** The time (seconds) that the rat stays in the open arm. **(E)** The percentage (%) of time rats stay in open arms. **(F)** Barnes Maze results, which assess animals’ learning and memory abilities. Representative activity trajectories of three groups of animals on the third day of the Barnes maze. The time taken by animals on Days 27, 28, and 29 to locate the target box is shown in panel **(G)**. A memory test was conducted on Day 30, with representative movement trajectories of each group of rats illustrated. The time spent in the target quadrant (upper right corner) is displayed in panel **(H)**. Data represent mean ± SE (*n* = 6–7). *P < 0.05 versus control group, #*P* < 0.05 versus EE group. Statistical significance was determined using One-way ANOVA analysis followed by the Tukey’s multiple comparisons or Two-way ANOVA analysis followed by the Tukey’s multiple comparisons.

On day 27, the elevated plus maze was conducted. As shown in Figure 3A, the movement trajectories of the three groups of rats in the elevated plus maze indicated that the EE group exhibited reduced exploratory activity in the open arms compared to the control group. The number of entries into the open arms, the percentage of entries, and the time spent in the open arms were all significantly decreased (*P* < 0.05, Figures 3B–E), suggesting that rats subjected to exhaustive exercise training displayed anxiety-like behavior. Interestingly, the MB group showed a significant increase in the number of entries, the percentage of entries, and the time spent in the open arms compared to the EE group (*P* < 0.05, Figures 3B–E), indicating that intranasal administration of MB can alleviate anxiety-like behavior induced by exhaustive exercise training.

To investigate the changes in spatial learning and memory abilities following exhaustive swimming training, the Barnes maze was conducted from day 27 to day 30. During the training trial (day 27–29), all three groups showed a reduction in the time taken to locate the hidden escape box. The representative search trajectories from the training trial (Day 29, Figure 3F) and the subsequent probe trial (Day 30) visually summarize the patterns of spatial learning and memory retention, respectively. Although the EE group required more time to locate the hidden escape box compared to the control group and the MB group (Figure 3G), the difference was not statistically significant (*P* > 0.05). These results suggest that the exhaustive exercise training paradigm used in this study may not cause spatial learning deficits in rats. Nevertheless, in the probe trial (day 30), the EE group spent significantly less time in the target quadrant compared to the control animals, indicating that exhaustive exercise induced memory deficits (*P* < 0.05, Figure 3H). Interestingly, this deficit was alleviated in the MB group, suggesting that MB treatment significantly improved spatial memory deficits following exhaustive exercise.

### 3.3 MB prevented hippocampal neuron loss and inhibits activation of apoptotic pathways in rats exposed to exhaustive exercise training

**FIGURE 4:**
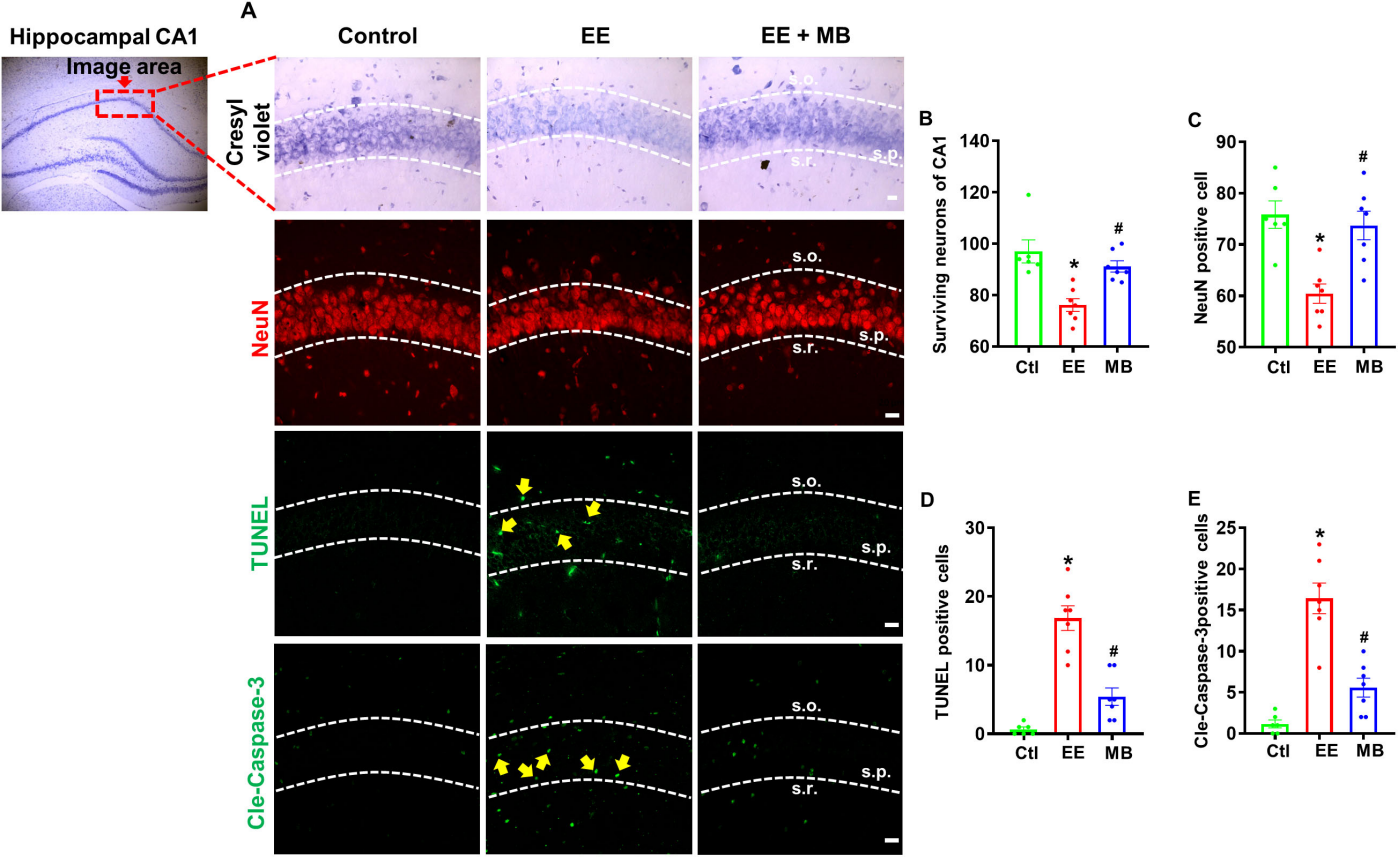
Methylene blue (MB) prevented hippocampal neuron loss and inhibited activation of apoptotic pathways in rats exposed to exhaustive exercise training. **(A)** Representative images of Cresyl violet staining, NeuN staining (red), and cleaved caspase-3 and TUNEL staining (green) in the hippocampal region after MB intervention. Surviving neurons of CA1 **(B)**, NeuN positive cell **(C)**, TUNEL **(D)**, and cleaved caspase-3 **(E)** were quantified and expressed as percentage changes compared to the respective control group. Data represent mean ± SE (*n* = 6–7). *P < 0.05 versus control group, #*P* < 0.05 versus EE group. The scale bar = 10 μm. Statistical significance was determined using One-way ANOVA analysis followed by the Tukey’s multiple comparisons.

To investigate whether exhaustive exercise training induces neuronal loss and whether this effect can be mitigated by MB treatment, Cresyl violet staining was used to quantify survival neurons in the hippocampal CA1 region. Cresyl violet has a high affinity for Nissl bodies in neurons and is widely used for observing the structure of neurons and Nissl bodies in neural tissue (Ahn et al., 2021). Additionally, neuronal nuclei protein (NeuN) is considered a reliable marker for mature neurons (Duan et al., 2016). As shown in Figure 4A, subjected to exhaustive swimming exhibited significant neuron loss in the hippocampal CA1 region, as evidenced by a reduction in NeuN-positive neurons and decreased neuronal survival detected by Cresyl violet staining. Compared to the EE group, MB intervention significantly prevented EE-induced neuronal loss, indicated by a marked increase in NeuN labeling in the hippocampal CA1 region. Quantitative analysis of surviving positive neurons further confirmed that MB notably reversed EE-induced neuronal loss (*P* < 0.05, Figures 4B, C).

To further elucidate whether MB can inhibit apoptosis resulting from exhaustive exercise, we performed immunofluorescence staining for cleaved caspase-3, a key executioner of apoptosis, and conducted a TUNEL assay to detect DNA fragmentation, a definitive hallmark of apoptotic cell death. As shown in Figure 4A and quantified in Figures 4D, E, EE significantly increased the immunoactivity of cleaved caspase-3 and TUNEL signal in the hippocampal CA1 region compared to the control group (*P* < 0.05). Notably, MB treatment significantly reduced the activities of both cleaved caspase-3 and TUNEL signal (*P* < 0.05).

### 3.4 MB prevented loss of striatal neurons and myelin protein in rats exposed to exhaustive exercise training

**FIGURE 5:**
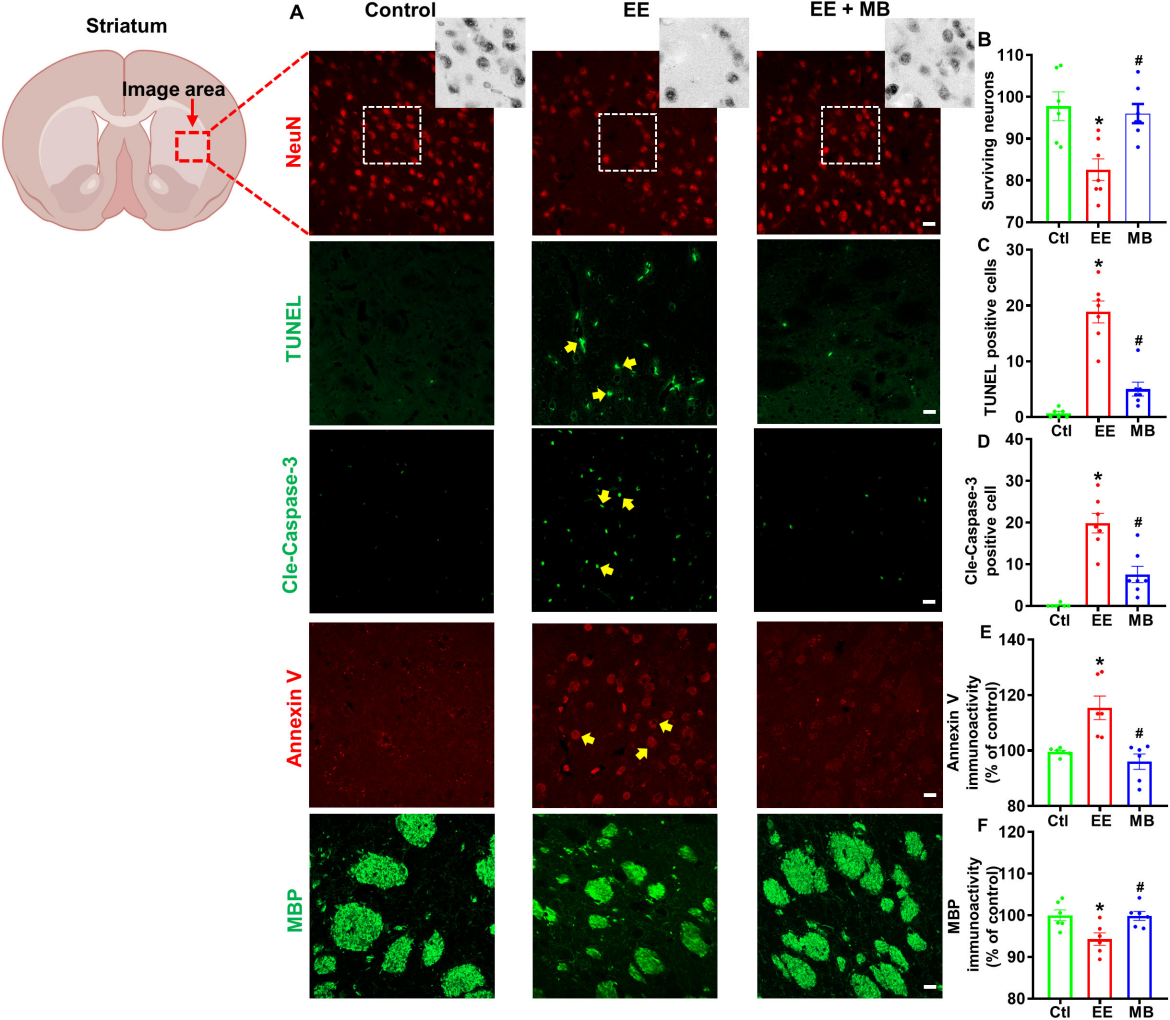
Methylene blue (MB) prevented loss of striatal neurons and myelin protein in rats exposed to exhaustive exercise training. **(A)** Representative images of NeuN staining (red), cleaved caspase-3, TUNEL staining (green), Annexin V staining (red) and MBP staining (green) in the striatum region after MB intervention. Number of surviving neurons **(B)**, TUNEL **(C)**, cleaved caspase-3 **(D)**, Annexin V **(E)** and MBP **(F)** were quantified and expressed as percentage changes compared to the respective control group. Data represent mean ± SE (*n* = 6–7). *P < 0.05 versus control group, #*P* < 0.05 versus EE group. The scale bar = 10 μm. Statistical significance was determined using One-way ANOVA analysis followed by the Tukey’s multiple comparisons.

To investigate the effects of intranasal MB administration on striatal neuron survival and myelin integrity in exercise-fatigued rats, we utilized neuronal marker NeuN, apoptotic protein marker Annexin V, and myelin protein marker MBP to stain and analyze brain sections of the rats. During apoptosis, the symmetry of the cell membrane is disrupted, causing phosphatidylserine (PS), normally located on the inner side of the membrane, to flip outward to the membrane’s exterior. Annexin V specifically binds to the exposed PS with high specificity, making it a classical marker for the early detection of apoptosis (Yagle et al., 2005; Brumatti et al., 2008). Myelin basic protein (MBP), the second most abundant protein in the CNS after proteolipid protein (PLP), is distributed on the cytoplasmic side of the myelin sheath, interacts with the cytoskeleton, and serves as an indicator of myelin structural functionality (Boggs, 2006; Haris et al., 2022). As shown in Figure 5A, striatal neurons in rats subjected to exhaustive swimming exhibited a significant loss of NeuN-positive neurons. Compared to animals in the EE group, MB treatment significantly reduced EE-induced neuronal loss, as evidenced by a marked increase in NeuN labeling in this region. We conducted a quantitative analysis of surviving neurons and confirmed that MB could significantly reverse neuronal loss caused by EE (*P* < 0.05, Figure 5B). To further elucidate the inhibitory effect of MB on EE-induced neuronal apoptosis, we assessed cleaved caspase-3, TUNEL signal, and Annexin V immunoactivity, which are involved in the apoptotic pathway. As shown in Figure 5A, EE significantly increased the immunoactivity of cleaved caspase-3, TUNEL signal, and Annexin V in the striatal region compared to the control group. MB treatment led to a significant reduction in the activity levels of these markers. Quantitative analyses of cleaved TUNEL signal, caspase-3, and Annexin V activity confirmed that MB suppressed the activation of the apoptotic pathway (*P* < 0.05, Figures 5C–E). Furthermore, as shown in Figure 5F, compared to control rats, EE resulted in a fragmented and dispersed distribution of striatal MBP with reduced fluorescence intensity. However, MB treatment effectively prevented the decrease in MBP expression in animals subjected to exhaustive swimming (*P* < 0.05).

### 3.5 MB treatment significantly inhibited EE-induced loss of dendritic and synaptic proteins

**FIGURE 6:**
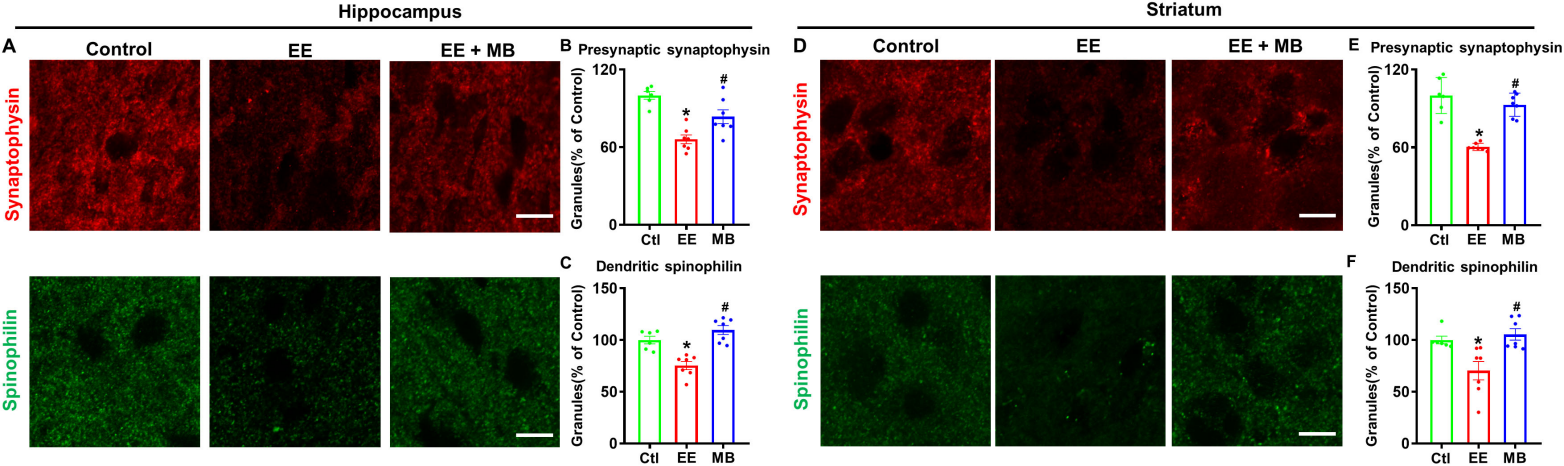
Methylene blue (MB) Treatment significantly inhibited EE-induced loss of dendritic and synaptic proteins. **(A)** Representative immunofluorescence images of the presynaptic (synaptophysin) and postsynaptic marker (spinophilin) in the hippocampal CA1 region, and the intensity of synaptophysin and spinophilin were quantified and expressed as percentage changes compared to the respective control group in panels **(B–D)** Representative immunofluorescence images of the presynaptic (synaptophysin) and postsynaptic marker (spinophilin) in the striatum region, and the intensity of synaptophysin and spinophilin were quantified and expressed as percentage changes compared to the respective control group in panels **(E,F)**. Data represent mean ± SE (*n* = 6–7). *P < 0.05 versus control group, #*P* < 0.05 versus EE group. The scale bar = 10 μm. Statistical significance was determined using One-way ANOVA analysis followed by the Tukey’s multiple comparisons.

Next, we examined expressions of spinophilin (a postsynaptic marker) and synaptophysin (a presynaptic marker) in the hippocampal and striatal regions. In the hippocampal region, as shown in Figure 6A, the levels of synaptophysin and spinophilin in EE rats were significantly reduced compared to control animals (synaptophysin: *P* < 0.0005, Figure 6B; spinophilin: *P* < 0.005, Figure 6C). Conversely, the reduction in synaptophysin and spinophilin observed in EE rats was significantly alleviated following MB treatment (synaptophysin: *P* < 0.05, Figure 6B; spinophilin: *P* < 0.0001, Figure 6C). Similarly, in the striatal region, as shown in Figure 6D, the levels of synaptophysin and spinophilin in the EE group were significantly decreased compared to the control group (synaptophysin: *P* < 0.0001, Figure 6E; spinophilin: *P* < 0.05, Figure 6F). This loss of dendritic and synaptic proteins was significantly rescued by MB treatment (synaptophysin: *P* < 0.0001, Figure 6E; spinophilin: *P* < 0.05, Figure 6F). These results indicate that MB provides effective therapeutic benefits against EE-induced synaptic atrophy.

### 3.6 MB suppressed EE-induced excessive activation of glial cells following exhaustive exercise

**FIGURE 7:**
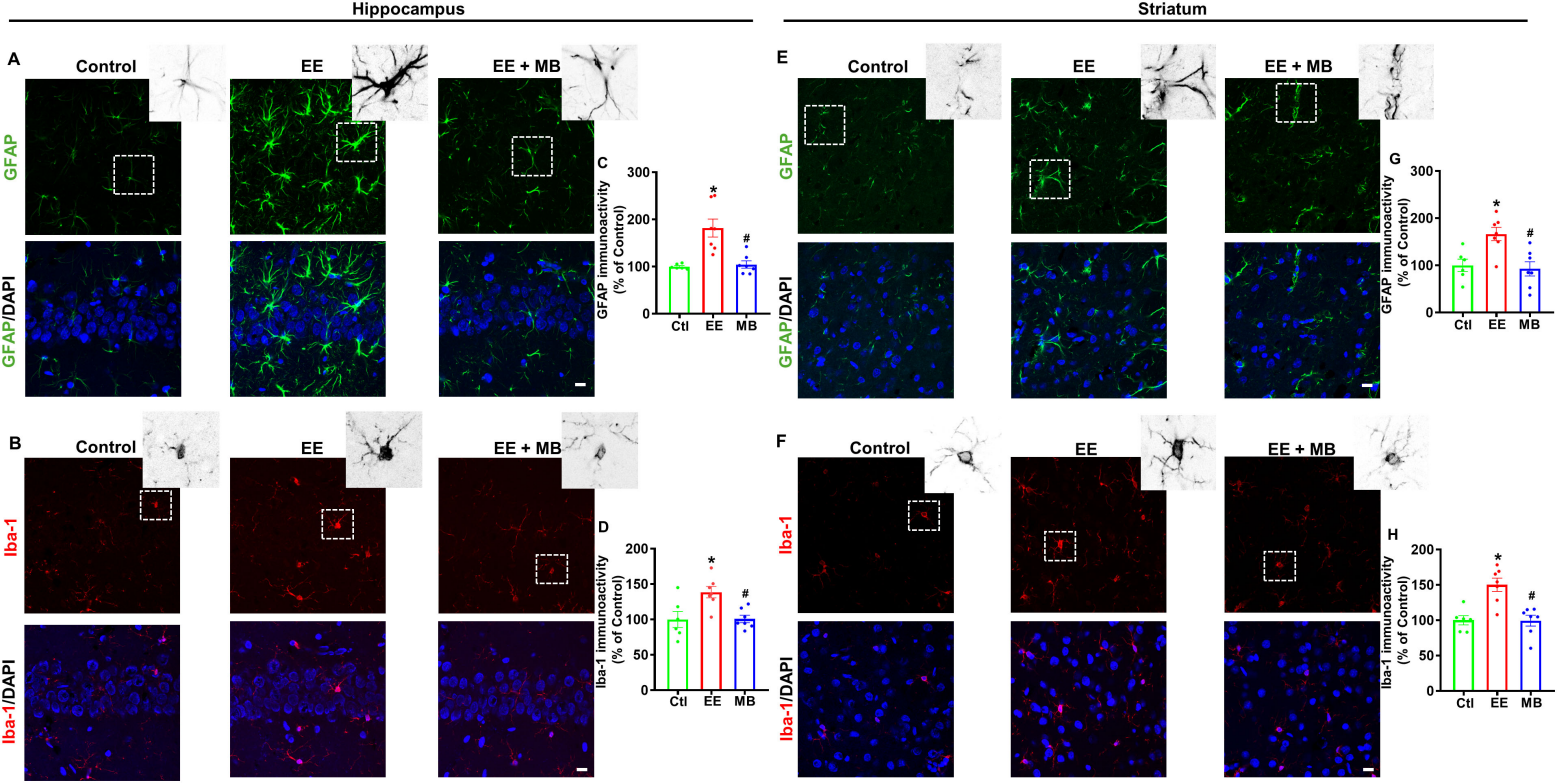
Methylene blue (MB) suppressed EE-induced excessive activation of glial cells following exhaustive exercise. Representative immunofluorescence images of GFAP **(A)** and Iba-1 **(B)** in the hippocampal CA1 region, and the immunoactivity of GFAP and Iba-1 were quantified and expressed as percentage changes compared to the respective control group in panels **(C,D)**. Representative immunofluorescence images of GFAP **(E)** and Iba-1 **(F)** in the striatum region, and the immunoactivity of GFAP and Iba-1 were quantified and expressed as percentage changes compared to the respective control group in panels **(G,H)**. Data represent mean ± SE (*n* = 6–7). *P < 0.05 versus control group, #*P* < 0.05 versus EE group. The scale bar = 10 μm. Statistical significance was determined using One-way ANOVA analysis followed by the Tukey’s multiple comparisons.

Excessive activation of glial cells can lead to further damage and apoptosis of neurons following injury (Speth et al., 2000; Choi et al., 2016). To explore the impact of MB intervention on glial cell activation in EE rats, we measured the fluorescence expression of GFAP (a classical marker of astrocytes) and Iba-1 (a marker of microglia/macrophages) in the hippocampus and striatum. As shown in Figure 7A, B, the fluorescence expression of GFAP and Iba-1 within the hippocampus was significantly increased after EE (GFAP: *P* < 0.0005, Figure 7C; Iba-1: *P* < 0.001, Figure 7D), indicating that EE-induced excessive glial activation. Conversely, animals treated with MB exhibited a significant reduction in the GFAP and Iba-1 fluorescent expression (GFAP: *P* < 0.0005, Figure 7C; Iba-1: *P* < 0.001, Figure 7D). Interestingly, a similar pattern was observed in the striatum, as showed in Figures 7E, F. A profound increase in GFAP and Iba-1 fluorescence expression was observed in the striatum following exhaustive exercise training (GFAP: *P* < 0.0005, Figure 7G; Iba-1: *P* < 0.001, Figure 7H). However, this increase in GFAP and Iba-1 expression was mitigated in animals treated with MB (GFAP: *P* < 0.0005, Figure 7G; Iba-1: *P* < 0.001, Figure 7H). In summary, EE induced excessive activation of glial cells in both the hippocampal and striatal regions, while MB significantly inhibited this effect.

### 3.7 MB treatment protected mitochondrial morphology following exhaustive exercise training

**FIGURE 8:**
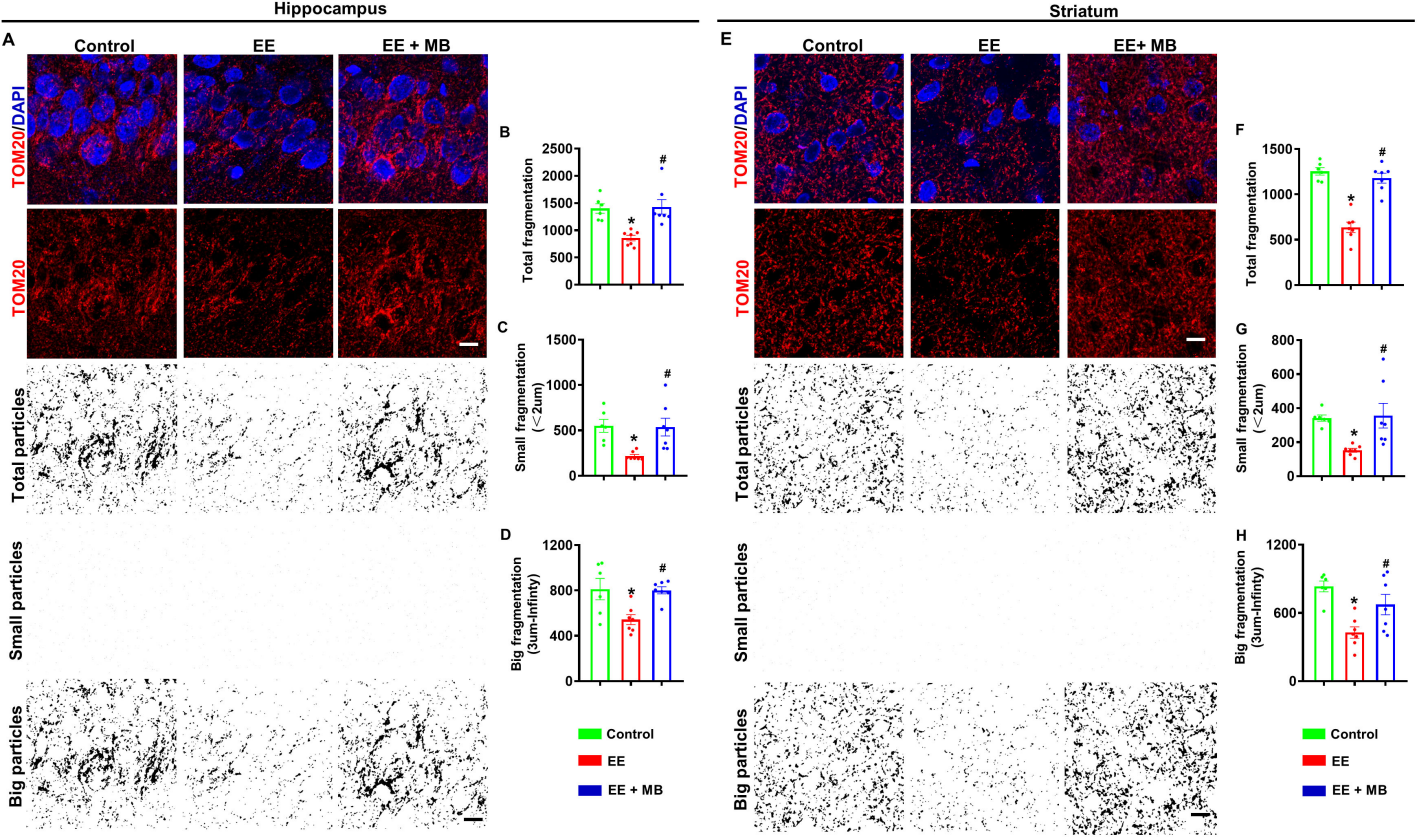
Methylene blue (MB) treatment protected mitochondrial morphology following exhaustive exercise training. **(A)** Representative confocal microscopy images of the hippocampus showing DAPI (blue) and Tom 20 (red) are shown in figure. The confocal images of Tom 20 were thresholded, filtered (median), and binarized using image J software. As shown in figure, mitochondrial segments were separated into total particles, small particles (size: 0–2 μm), and big particles (size: 3 μm- infinity). The analysis of mitochondrial fragments involved counting, as shown in panels **(B–D)**. The total count of mitochondrial fragments **(B)**, the count of small mitochondrial fragments **(C)**, and the large mitochondrial structures **(D)** are normalized to the total mitochondrial area. **(E)** Representative confocal microscopy images of the striatum region showing DAPI (blue) and Tom 20 (red) are shown in figure. The confocal images of Tom 20 were thresholded, filtered (median), and binarized using image J software. As shown in figure, mitochondrial segments were separated into total particles, small particles (size: 0–2 μm), and big particles (size: 3 μm- infinity). The analysis of mitochondrial fragments involved counting, as shown in panels **(F–H)**. The total count of mitochondrial fragments **(F)**, the count of small mitochondrial fragments **(G)**, and the large mitochondrial structures **(H)** are normalized to the total mitochondrial area. Data represent mean ± SE (*n* = 6–7). *P < 0.05 versus control group, #*P* < 0.05 versus EE group. The scale bar = 10 μm. Statistical significance was determined using One-way ANOVA analysis followed by the Tukey’s multiple comparisons.

We next evaluated the effects of MB treatment on mitochondrial morphology following EE in the hippocampus and striatum regions. In the hippocampus, as shown in Figure 8A, animals in the EE group exhibit increased mitochondrial fragmentation and disrupted morphology compared to normal animals and those treated with MB. To further define and analyze these changes, we processed the TOM20 fluorescence images using ImageJ software by applying thresholding and binarization. Quantitative analyses of total mitochondrial particles, small particles, and large particles are shown in Figures 8B–D. The results indicate that the reduction in mitochondrial structures was significantly greater in the EE group compared to the control group, whereas MB treatment attenuated mitochondrial fragmentation (*P* < 0.05). Furthermore, similar results were observed in the striatum, as illustrated in Figure 8E. The TOM20 signal in the control and MB groups was evenly distributed with intact structures, whereas the EE group displayed diminished TOM20 signal and pronounced structural fragmentation. Further quantitative analysis in Figures 8F–H revealed that MB-treated EE rats exhibited more intact TOM20 particles, showing no significant difference from the control group. In contrast, EE rats showed notable mitochondrial fragmentation, with total and fragmented particles significantly decreased compared to the control and MB groups (*P* < 0.05). These findings indicate that MB offers protective effects against exhaustive exercise-induced mitochondrial damage.

## 4 Discussion

The adverse effects of prolonged exhaustive exercise have been investigated for many years, but research has primarily focused on its peripheral impact. In recent years, the adverse neurological consequences of prolonged exhaustive exercise have gained increasing research interest. Converging evidence suggests that prolonged exhaustive exercise may lead to slowed reaction times, impaired learning and memory, motor impairments, heightened anxiety, and other emotional disturbances (Ament and Verkerke, 2009; Xie et al., 2024). Currently, there are various anti-fatigue methods, each with its own advantages and disadvantages. For example, pre-cooling therapy, cold water immersion, electrical stimulation, and laser therapy have shown significant effectiveness but require external equipment, which can be costly. Nutritional supplementation, widely used in the sports industry, involves the intake of high-protein, high-carbohydrate foods, and energy supplements shortly after exercise. While this approach has demonstrated clear anti-fatigue benefits, it requires careful attention to dosage, consumption methods, and potential side effects (Nedelec et al., 2013; Querido et al., 2022). However, the above-mentioned interventions are often limited by the need for specialized equipment and narrow windows of application, and there is no emphasis on the protection of the CNS. Therefore, identifying strategies to promote rapid recovery after exhaustive exercise and prevent neural damage is critically important.

In this study, we investigated the effects of intranasal MB administration on neuronal apoptosis, synaptic structure damage, glial cell activation, and mitochondrial dynamics in the hippocampus and striatum of rats following exhaustive exercise training. Ten consecutive days of exhaustive swimming exercise represent a highly stressful state for the animals. High-intensity exercise has effects on the brain similar to ischemia/reperfusion, leading to central nervous oxidative stress and tissue necrosis (Wu et al., 2012; Peng et al., 2021). Moreover, studies have shown that physical activity alters the number of neurons in rats, involving the regulation of cell proliferation and apoptosis (Lattanzi et al., 2022). Related research has indicated that high-intensity exercises such as marathons and triathlons can trigger strong immune/inflammatory responses, causing vascular damage, respiratory diseases, and gastrointestinal disorders. Additionally, gene expression differences related to metabolism, DNA methylation, apoptosis, and brain function regulation can be detected shortly after high-intensity exercise (Hanke et al., 2010; Castell et al., 2019). Furthermore, overtraining can lead to increased cortisol levels in the blood of animals (da Rocha et al., 2017). For these reasons, metabolic imbalances and excessive oxidative stress responses can result in mitochondrial dysfunction, further leading to neuronal death and increasing the risk of developing mental disorders (Ren et al., 2023). Existing research indicates that MB plays a critical role in neuroprotection through mechanisms such as inhibiting oxidative stress, improving mitochondrial dysfunction, suppressing inflammatory responses, and regulating the expression of endocrine hormones like dopamine (Ou et al., 2023).

Methylene blue is characterized by its low dosage requirement, rapid efficacy, minimal side effects, and affordable price (Wu et al., 2024). Research has shown that MB solution provides significant neuroprotective effects in neonatal SD rat models of hypoxia-ischemia, with no notable side effects (Zhang et al., 2020). In this study, MB solution was used as an intervention, administered intranasally to address exhaustive exercise-induced neurological deficits. The present study observed that MB mitigated mitochondrial morphological changes induced by prolonged exhaustive exercise training, inhibiting apoptotic pathway activation and glial cell activation. This helped protect neuronal survival in the hippocampus and striatum regions, demonstrating its neuroprotective effects. Therefore, this study provides evidence supporting MB as a potential strategy to counteract neuronal deficits induced by exhaustive exercise. Many studies have shown that exhaustive exercise can lead to a decrease in the number of hippocampal neurons, and changes in microstructure (Chen et al., 2009; Liu et al., 2024). This research corroborates previous findings by observing, through cresyl violet staining, that neurons in the CA1 region of the hippocampus in rats appear smaller in volume, more sparsely distributed, and show signs of nuclear condensation, nuclear shrinkage, and blurred Nissl bodies. These findings suggest that high-intensity exercise causes cell necrosis and apoptosis in the hippocampal region. After 10 days of intranasal administration of MB, improvements were observed in the cresyl violet staining in the hippocampal CA1 region, with neuron volume returning to normal and distribution becoming denser. We further examined key markers of apoptosis and found that exhaustive exercise (EE) resulted in increased cleaved caspase-3 immunoreactivity and TUNEL signal in the hippocampal CA1 region. Notably, intranasal MB administration mitigated this neuronal apoptosis. This cellular-level protection was associated with functional improvements; in the Barnes maze, rats in the EE group exhibited memory deficits that were alleviated in the MB group. This cognitive improvement may be related to the restoration of synaptic structures in the hippocampal CA1 region, leading to enhanced synaptic plasticity.

The striatum, as a key area for motor regulation, can enhance neural activity and synaptic plasticity in the striatum with appropriate exercise intensity. However, intense or high load exercise has a negative impact on the survival of striatal neurons and synaptic structure (Tamakoshi et al., 2014; Wang Z. et al., 2019). This study examined the expression of neuronal nuclear protein NeuN, MBP, synaptophysin, and spinophilin in the striatum. The findings revealed that exhaustive exercise led to pathological alterations, including the loss of dorsolateral striatal neurons, reduced myelin sheath, and diminished spinophilin expression in rats. Synaptic plasticity is closely linked to learning and memory, and since the striatum is involved in motor regulation, synaptic function is likely associated with motor ability and performance in rats (Grillner et al., 2005; Chang et al., 2006; Kreitzer and Malenka, 2008). During the experiment, we observed a stark contrast in physical endurance. Rats in the exhaustive exercise (EE) group were progressively unable to sustain swimming, indicative of severe fatigue. In contrast, rats receiving intranasal MB showed markedly improved performance. To investigate a potential cellular basis for this exercise-induced central fatigue, we examined key apoptotic markers in the striatum. Analysis revealed that compared to the control group, the EE group exhibited a significant increase in the levels of cleaved caspase-3, as well as in TUNEL and Annexin V signals. Notably, 10 days of intranasal MB administration robustly reversed these pro-apoptotic changes.

Multiple research studies have shown that exhaustive exercise may disrupt the immune system, leading to immunosuppression and an excessive inflammatory response in athletes. These responses include the expression of pro-inflammatory factors such as TNF-α and IL-6, which can further contribute to neurological disorders or negatively affect normal physiological states (Vargas and Marino, 2014; Clark and Mach, 2016; Li et al., 2019). Scientific studies indicate that abnormal activation of astrocytes and microglia not only produces more pro-inflammatory factors, such as IL-1β and IL-6, causing more severe inflammatory responses, but also damages the CNS, further contributing to cognitive deficits in mice (Wang G. et al., 2019; Wang W. et al., 2024). Interestingly, exhaustive swimming exercises significantly increase the activation levels of GFAP and Iba-1 in the hippocampus and striatum, which can be reverse by MB treatment.

Mitochondria are essential organelles within cells, often referred to as the cell’s powerhouses, as they generate ATP through oxidative phosphorylation to provide energy for cellular activities (Nunnari and Suomalainen, 2012). Additionally, mitochondria participate in various metabolic processes, including glycolysis, amino acid metabolism, and fatty acid oxidation (Kelly and Pearce, 2020). Over the past decade, mitochondrial dysfunction has been closely linked to various CNS disorders (Morato et al., 2014; Wang H. et al., 2024). Studies have shown that restoring mitochondrial dynamics and inhibiting mitochondrial dysfunction to recover energy metabolism in different brain regions can significantly improve behavioral deficits in animal models of Alzheimer’s disease and Parkinson’s disease (Li et al., 2023). Previous research reports indicate that excessive exercise may alter the permeability of the mitochondrial inner membrane, with damaged mitochondria leading to excessive fragmentation, impaired mitochondrial enzyme complexes, reduced ATP levels, and the release of cytochrome c into the cytoplasm–an early apoptotic event during overexertion (Wang et al., 2023). Following the release of cytochrome c from mitochondria, caspase-3 and caspase-9 are activated, forming complexes with cytochrome c, thereby promoting subsequent apoptotic signaling pathways (Strobel et al., 2011; Zhang et al., 2022). Furthermore, studies have demonstrated that acute or high-intensity exercise can significantly increase apoptotic T cells and lymphocyte subsets CD4 and CD8 cells in human blood, with early apoptotic markers such as Annexin V, caspase-3, and caspase-9 activation further confirming that intense acute exercise accelerates apoptosis (Navalta et al., 2013; Palmowski et al., 2020). The findings of this study indicate that MB improves neural damage by inhibiting mitochondrial fragmentation in the hippocampus and striatum of rats and downregulating the expression levels of cleaved caspase-3, TUNEL and Annexin V signals. This further supports the potential of MB as a therapeutic agent for treating hippocampal and striatal neural damage caused by excessive exercise.

Overall, intranasal administration of MB demonstrated neuroprotective effects in a swim-exhaustion rat model. Although the detailed mechanisms of MB’s neuroprotective effects warrant further investigation, our findings suggest that MB can alleviate neuronal damage in the hippocampus and striatum while improving the physical condition of the exhausted rat model. Furthermore, this study did not measure oxidative reactions, inflammation, or hormone levels in the brain and plasma following intranasal MB administration. Future studies exploring the relationship between oxidative stress in the CNS, dosage, and treatment windows after intranasal MB administration would be particularly interesting. Additionally, the structural integrity of mitochondria relies on the regulation of mitochondrial fusion proteins. Mitochondrial fusion proteins Mfn1 and Mfn2 primarily mediate outer membrane fusion and play critical roles in maintaining normal mitochondrial morphology and function. Mitochondrial fusion can inhibit autophagy and protect mitochondrial functions by removing damaged mitochondrial proteins, highlighting the necessity for further investigation into mitochondrial-related proteins (Chen et al., 2003, 2011; Chan, 2012). Future research should also consider the comprehensive effects of MB supplementation prior to exercise on extreme physical performance.

A key consideration is the nature of the exercise model itself. By omitting a gradual adaptation period, our protocol more closely represents an acute stress paradigm rather than a state of physiological fatigue from adapted exercise. This distinction is critical, as the observed cognitive deficits were likely driven by this acute stress, meaning the benefits of MB may stem from its known neuroprotective and stress-mitigating properties, rather than a direct role in physiological recovery. Future studies employing an adapted training protocol are therefore essential to disentangle these two potential mechanisms.

Further investigations are also warranted to fully elucidate MB’s effects. This should include measuring key biomarkers (oxidative stress, inflammation, hormone levels) and examining mitochondrial fusion proteins like Mfn1 and Mfn2. Exploring different dosages, treatment windows, and the effects of pre-exercise supplementation would also provide a more comprehensive understanding of MB’s therapeutic potential in this context.

## Data Availability

The original contributions presented in this study are included in this article/supplementary material, further inquiries can be directed to the corresponding authors.
